# Combined Antibiotic and Photodynamic Therapies in *Pseudomonas aeruginosa*: From Synergy to Antagonism

**DOI:** 10.3390/antibiotics13121111

**Published:** 2024-11-21

**Authors:** Amanda C. Zangirolami, Koteswara Rao Yerra, Vladislav V. Yakovlev, Kate C. Blanco, Vanderlei S. Bagnato

**Affiliations:** 1 Biomedical Engineering, Texas A&M University, College Station, TX 77843, USA; zangiamanda@tamu.edu (A.C.Z.); ykrao@tamu.edu (K.R.Y.); yakovlev@tamu.edu (V.V.Y.); kateblanco@ifsc.usp.br (K.C.B.); 2 São Carlos Institute of Physics, University of São Paulo, São Carlos 13566-590, São Paulo, Brazil

**Keywords:** *Pseudomonas aeruginosa*, photodynamic therapy, antibiotic synergy, dosage optimization, antibiotic resistance

## Abstract

**Background:** Antibiotics remain the most effective option for combating infections. However, the situation has shifted from ideal to concerning, as bacterial resistance to antibiotics is increasing in both prevalence and strength. **Objectives**: This study explores the synergistic/antagonistic potential of combining antibiotic and photodynamic therapy (PDT) against *Pseudomonas aeruginosa*. **Methods**: We conducted in vitro experiments to observe the effect of the sequential application of antibiotics and photodynamic therapy with a time interval between them. The antibiotics used were ciprofloxacin, ceftriaxone, and gentamicin, and Photodithazine was employed as the photosensitizer, with the PDT performed at different light doses of 660 nm radiation. **Results**: The combined effect was highly dependent on the antibiotic. While for gentamicin, the combination of antibiotic and PDT treatment was always synergistic, for ciprofloxacin, it could be severely antagonistic. Each antibiotic exhibited a distinctive pattern of interaction with PDT. Gentamicin resulted in the largest enhancement in bactericidal activity combined with PDT, requiring lower antibiotic concentrations to achieve significant bacterial reduction. Ceftriaxone’s bactericidal action was less influenced by PDT intensity, maintaining a stable efficacy regardless of different PDT dosages. Conversely, the outcome of ciprofloxacin was highly dependent on the antibiotic concentration changing from synergic to antagonistic action. **Conclusions**: The findings advocate for the development of treatment protocols that combine antibiotics and PDT and necessitate the establishment of the criterion for the dosage and periodicity of administration of such combination protocols. The demonstrated results open the doors wide to new applications and opportunities to combat infectious diseases through the combined use of photodynamic therapy and antibiotics.

## 1. Introduction

Antimicrobial treatment strategies that can be used in conjunction with antibiotics are of great significance in medicine, especially in the context of increasing bacterial resistance [1]. The use of compounds that act synergistically with antibiotics can substantially improve the effectiveness of treatments, enabling the use of lower doses of antibiotics and, consequently, reducing the risk of developing resistance [1]. Such a synergistic effect can not only broaden the spectrum of activity of existing antibiotics against resistant strains but also revitalize the use of antibiotics that have become less effective over time [2]. Therefore, agents that can be co-administered with antibiotics are crucial for combating bacterial infections and minimizing the impact of antimicrobial resistance on global public health [3]. Combined therapy that uses antibiotics together with photosensitizers is a promising strategy that is widely described in the scientific literature, especially in the field of photodynamic therapy [4]. This therapeutic strategy used as a complementary approach exploits the ability of photosensitizers, which are capable of producing reactive oxygen species (ROS) when activated by light at a specific wavelength, to induce the cell death of pathogens [5]. When used in conjunction with antibiotics, photosensitizers can increase antimicrobial efficacy through multiple mechanisms of action [6]. This synergy may be particularly valuable against multidrug-resistant pathogens, where antibiotics alone may be ineffective. The combination of photosensitizers with antibiotics not only enhances the antimicrobial effect by producing ROS that damage vital cellular structures of the pathogens but can also help reduce the concentration of antibiotics necessary to achieve the desired therapeutic effect, thus minimizing the risks of toxicity and development of resistance [5]. However, a notable challenge in the clinical implementation of these therapeutic combinations is the lack of accurate data on optimal dosing. Although the scientific literature presents numerous studies that demonstrate the synergistic potential of certain drug combinations, there is often a significant gap regarding defining dosages that optimize therapeutic efficacy while minimizing the risks of toxicity and adverse effects generated at very high concentrations [7]. This gap exists because determining optimal dosages for drug combinations is complex due to variable pharmacokinetic and pharmacodynamic interactions between the drugs involved [8]. Synergy can vary significantly depending on multiple factors, including the proportion of drugs, the route of administration, the relative administration time between the agents, and specific patient characteristics, such as age, weight, renal and hepatic function, and the presence of other medical conditions [8].

The increase in antibiotic dosages for the treatment of infections caused by *Pseudomonas aeruginosa* reflects the growing concern about antimicrobial resistance presented by this bacterium [9]. *P. aeruginosa* is known for its ability to develop resistance to multiple antibiotics, making it a difficult-to-treat pathogen, especially in hospital settings, where it can cause serious and even fatal infections in immunocompromised patients. Adaptation to antibiotic mechanisms of action and the ability to form biofilms contribute to the resilience of *P. aeruginosa*. Optimizing antibiotic therapy against *P. aeruginosa* also involves the use of combination therapies, where two or more antibiotics are used simultaneously to exploit synergistic effects and reduce the likelihood of resistance development [10]. Furthermore, the pharmacodynamics and pharmacokinetics of antibiotics are carefully considered to adjust dosages and administration intervals, maximizing efficacy while minimizing adverse effects [11]. Therefore, while evidence of synergy between drug combinations opens promising avenues for more effective and safer therapies, determining optimal dosages for these combinations remains a significant challenge [12]. The motivation for this research comes from the need for further optimization in antibiotic dosing regimens, ensuring that the therapeutic potential of synergistic combinations can be fully realized in clinical practice [13]. The time dependence of the antibiotic effect and the use of sequential, timely, correct applications of combined therapies are hypothesized to be important and are the focus of this research study. In this work, we performed a proof-of-principle experiment to demonstrate that the time sequence of the application of antibiotics and photodynamic therapy may be an important issue in developing the correct protocols to optimize treatment.

## 2. Results and Discussion

In a control experiment where light was not applied, all antibiotics acted on the microorganisms within 6 h of application. In ascending order, the effect of ciprofloxacin (CIP) was greater than the effect of ceftriaxone (CEF), which, in turn, was greater than the effect of gentamicin (GEN). Table 1 shows the main properties of each antibiotic used in this experiment.

**Figure 1:**
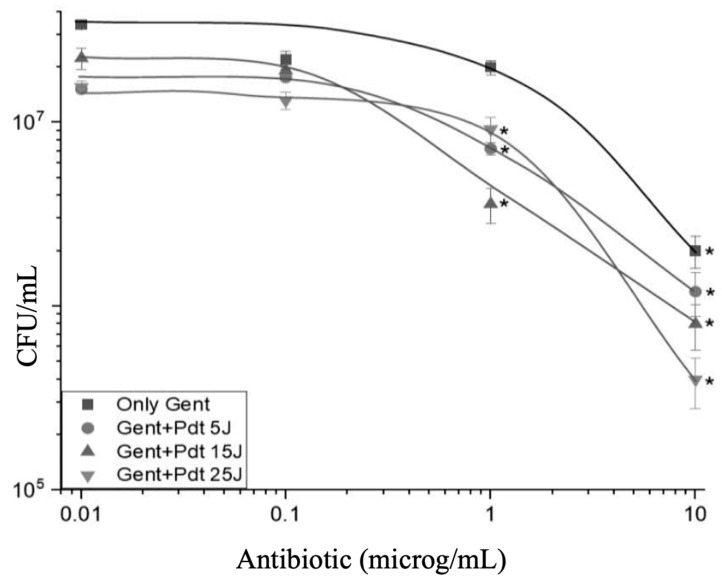
The combined effect of GEN and PDT on the viability of *P. aeruginosa*. The graph shows the count of colony-forming units (CFU/mL) as a function of GEN concentration (µg/mL) under different energy doses in PDT: 0 J/cm^2^ (■), 5 J/cm^2^ (●), 15 J/cm^2^ (▲), and 25 J/cm^2^ (▼). Statistical differences (indicated by an asterisk) was calculated by comparing each treated group (GEN + PDT) to the “only GEN” control group for each concentration level. Additionally, comparisons were made between specific time points to assess the influence of PDT doses over time. Error bars indicate standard deviation across replicates.

Figure 1 shows the microbial strain’s viability reduction under different combined concentrations of antibiotic and doses of light for the PDT involving the antibiotic (GEN). Analysis of the data reveals a decrease in colony-forming units (CFU) of *P. aeruginosa* as the antibiotic concentration increases, as evidenced by the descending curves corresponding to increasing doses (from 0 to 25 J/cm^2^). The curves exhibit a decay behavior, suggesting that higher doses of PDT enhance the antibiotic’s effect, leading to a more pronounced reduction in bacterial viability. That occurs for all the employed conditions in this study, suggesting a synergistic effect of both treatment components under the employed conditions.

In Figure 1, it is observed that 1 µg/mL of gentamicin combined with PDT using 25 J/cm^2^ resulted in a lower microbial reduction than other concentrations, but when we increased it to 10 µg/mL, the bactericidal effect was stronger, attributed to a concentration-dependent response in the combined therapy. At low concentrations, gentamicin may not have reached sufficient intracellular accumulation and consequently generated reactive oxygen species (ROS) by PDT, which led to bacterial death. At higher concentrations, the antibiotic reached levels at which antimicrobial therapeutic synergy with PDT occurred; probably, the ROS generated by PDT increased permeability at the cell membrane level, which facilitated the absorption of gentamycin and thus amplified its efficacy. Therefore, we observed an interaction between gentamicin and PDT sensitive to the concentration of the antibiotic and, therefore, describe the importance of optimizing both parameters for combined therapeutic efficacy.

**Figure 2:**
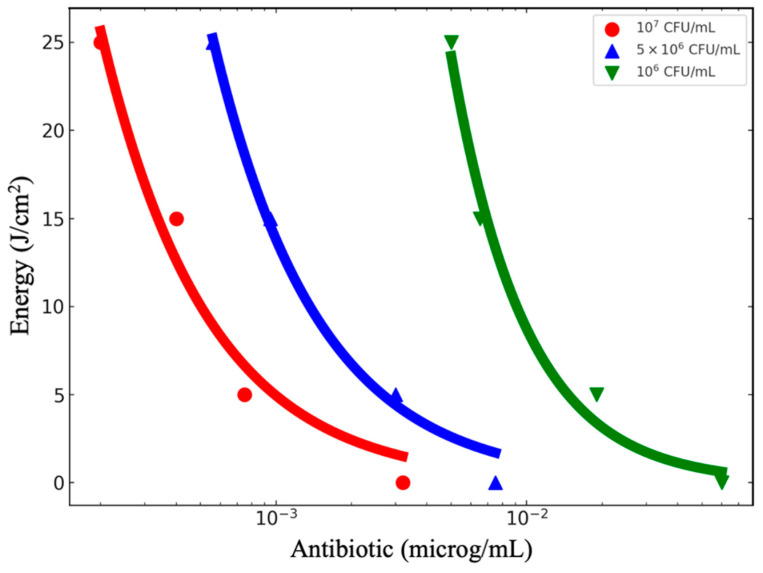
Equivalence plot between PDT under the described conditions and the concentration of GEN. Starting at about 2 × 10^7^ cells/mL, we have chosen three different microbicidal effects achievable with the photodynamic-antibiotic combination: 50% (red circles), 75% (blue triangles), and 95% (green inverted triangles) reduction in CFU/mL. The curves represent the energy and antibiotic concentration combinations necessary to reach each microbicidal effect under the experimental conditions.

GEN is particularly effective against *P. aeruginosa*, a Gram-negative bacterium that is notoriously resistant to many antibiotics due to its protective outer membrane and intrinsic resistance mechanisms [14]. GEN impairs bacterial growth and leads to cell death. It is particularly effective against Gram-negative bacteria as it is an aminoglycoside antibiotic that primarily inhibits bacterial protein synthesis [15]. Its mechanism of action involves irreversible binding to the bacterial ribosome (30S subunit), causing errors in mRNA reading and resulting in the production of defective or non-functional proteins [16]. The synergistic effect observed in the combination of GEN and PDT arises from the two treatments targeting distinct yet complementary cellular sites. While GEN acts on the ribosome, interrupting protein synthesis, PDT inflicts physical damage on the cellular structure, compromising membrane integrity and intracellular components [17]. These actions cause cellular stress that exceeds the bacteria’s ability to respond and repair, leading to a significantly higher death rate than observed with either treatment alone [18]. This synergistic effect induces irreparable damage to multiple targets within the bacterial cell. PDT enhances the action of GEN by increasing cell membrane permeability, facilitating antibiotic uptake, and amplifying its efficacy. Moreover, the reactive oxygen species (ROS) generated by PDT exacerbate oxidative stress, accelerating cellular dysfunction and bacterial death, which is evident in the greater reduction of bacterial counts in the combined doses. To be more specific with the synergic effect, there is an equivalence between the effect of antibiotics and the effect of PDT. Suppose one fixes the quantity of microorganisms to be eliminated with the combined action. In that case, we can determine the necessary combined doses of antibiotic and light that will promote the desired result. In Figure 2, we have selected a few microbicide effects and represented them in a plot of GEN concentration and light dose that will result from it. All the experiments start with the same microorganism concentration in the beginning, which was (2–2.5) ×10^7^ CFU/mL, obtained by the control in each experiment.

**Figure 3:**
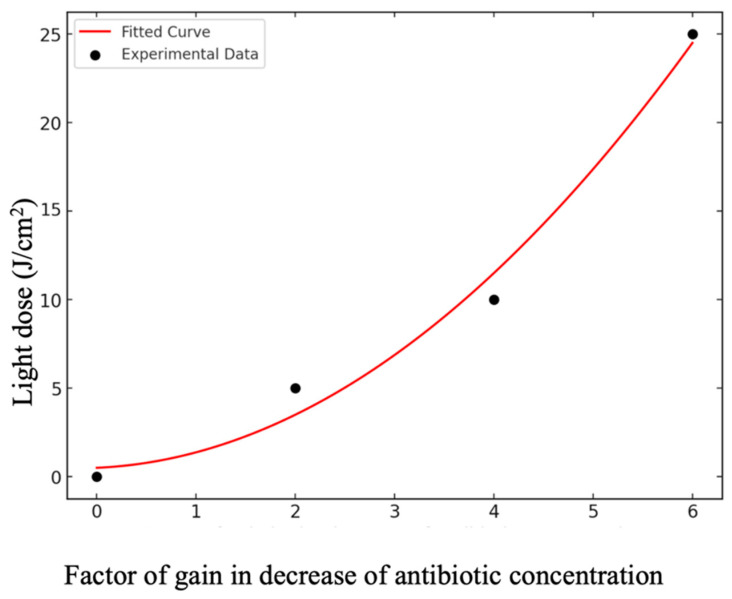
Relationship between the photodynamic light dose (J/cm^2^) and the factor of reduction in antibiotic concentration required to achieve the same antimicrobial effect. The plot shows the diminishing returns with increasing light dose, where higher doses result in smaller incremental reductions in antibiotic concentration, indicating a saturation trend. The red curve represents a mathematical fit to the experimental data points (black dots), highlighting the relationship between the photodynamic dose and antibiotic reduction efficiency.

If no photodynamic action is present (light dose = 0), a higher concentration of antibiotic GEN is necessary. On the other hand, as a dose of photodynamic therapy is gradually introduced, the dose of antibiotic decreases, as displayed in Figure 2. It can also be seen that for each additional amount of photodynamic action expressed in the form of deposited energy density, the required dose of antibiotic expressed in the form of its concentration decreases. The gain in antibiotic concentration reduction with the added photodynamic dose in the microbicide effect is shown in Figure 3.

As the light dose increases, the factor of reductions on the necessity of antibiotics increases. The format of the curve indicates that the gain per J/cm^2^ added decreases, indicating that increasing indefinitely the quantity of photodynamic dose does not contribute accordingly. There seems to be a range of good gains.

**Figure 4:**
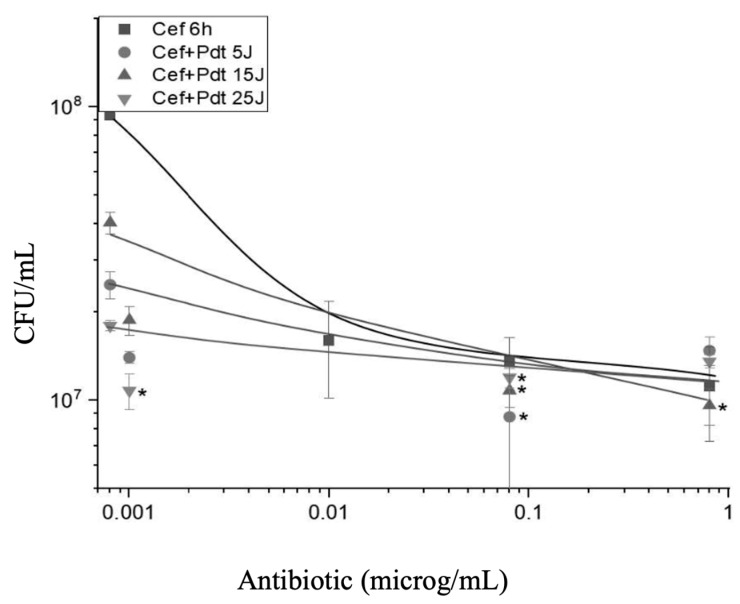
The combined effect of CEF and PDT on the viability of *P. aeruginosa*. The graph shows the count of colony-forming units (CFU/mL) as a function of CEF concentration (µg/mL) under different energy doses in PDT: 0 J/cm^2^ (■), 5 J/cm^2^ (●), 15 J/cm^2^ (▲), and 25 J/cm^2^ (▼). Statistical differences (indicated by an asterisk) was evaluated by comparing each treated group (CEF + PDT) with the “CEF 6 h” control group across the different concentrations. Additionally, comparisons were made between each time point to determine the impact of increased PDT doses on bacterial viability. Error bars represent the standard deviation from triplicate experiments.

Figure 4 shows the viability of *P. aeruginosa* (measured in CFU/mL) as a function of ceftriaxone (CEF) concentration (µg/mL) and different doses of PDT represented by 0, 5, 15, and 25 J/cm^2^. The graphs for CEF show that, in general, the PDT improved the action of the antibiotic in a very modest way. The variation achieved for this antibiotic, even at high concentrations, is very small. However, consistently, the presence of PDT improves the action of the antibiotic in high concentrations. The higher dose of PDT appears to consistently lead to appreciably better results. Despite this, PDT is of little significance in addition to antibiotics, which act modestly on microorganisms. An important observation is that the result when the combination photodynamic–CEF was performed is quite different for the equivalent situation with GEN. In Figure 4, the 6 h time point represents the duration of antibiotic incubation prior to PDT. During this time, the antibiotics had time to interact with Pseudomonas aeruginosa during its multiplication, allowing their antimicrobial effects to manifest before PDT treatment. This incubation time was chosen to achieve peak antimicrobial effect, establishing a consistent basis for evaluating the synergistic or antagonistic effects of PDT subsequent to antibiotics.

**Figure 5:**
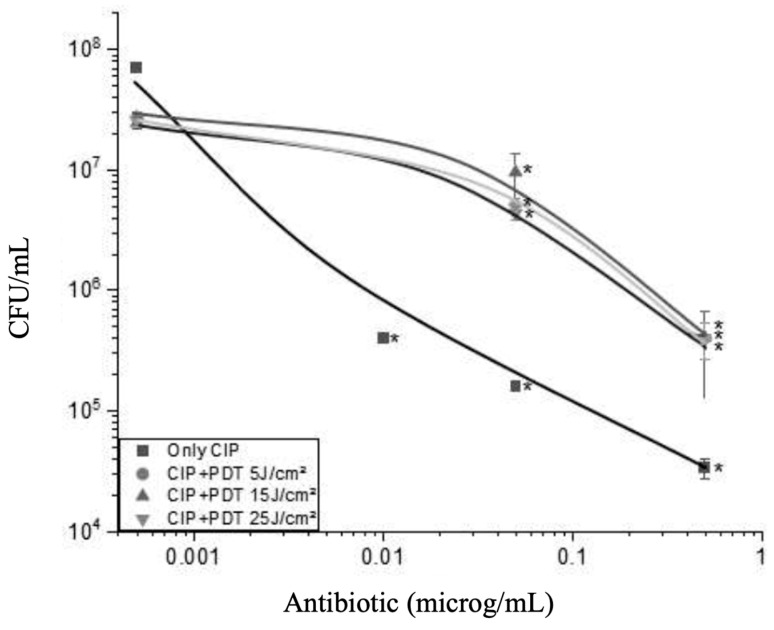
Evaluation of the combined effect of an antibiotic CIP and PDT on the viability of *P. aeruginosa*. The graph illustrates the colony-forming unit count (CFU/mL) as a function of the antibiotic concentration (µg/mL) under different energy doses in PDT: 0 J/cm^2^ (■), 5 J/cm^2^ (●), 15 J/cm^2^ (▲), and 25 J/cm^2^ (▼). * Statistical differences (indicated by an asterisk) was assessed by comparing each PDT-treated group (CIP + PDT) to the “Only CIP” control group at corresponding concentrations using a one-way ANOVA followed by Tukey’s test (*p* < 0.05). Error bars represent the standard deviation of triplicate measurements.

Figure 5 shows the viability of *P. aeruginosa* (in CFU/mL) as a function of the concentration of the antibiotic ciprofloxacin (CIP) combined with doses of PDT of 0, 5, 15, and 25 J/cm^2^. As the concentration of CIP increases, bacterial viability decreases more sharply, especially when combined with higher doses of PDT. CIP is an antibiotic of the fluoroquinolone class that acts by inhibiting the enzymes DNA gyrase and topoisomerase IV, essential for the replication, transcription, and repair of bacterial DNA. This inhibition leads to the accumulation of unrepaired DNA breaks, resulting in the interruption of DNA synthesis and cell death [19]. The graph shows that the efficacy of CIP increases with its concentration, especially in the absence of PDT (0 J/cm^2^).

In region I, which corresponds to a concentration of antibiotic CIP lower than 0.01 µ/mL, a marked reduction in bacterial viability is observed when combined with increasing doses of PDT. This behavior could indicate a marked synergistic effect between the two treatments in this concentration range. The synergy is attributed to the increase in cell permeability induced by PDT, which facilitates the entry of CIP, amplifying its bactericidal effect. For this region, increasing the dose seems to lead to better results. This region highlights the efficacy of the combination of therapies even at low doses of the antibiotic, showing that PDT can potentiate the action of CIP, making it effective at concentrations where its effect would normally be limited.

The synergy between PDT and CIP is most pronounced at moderate to high doses of PDT, where accumulated damage overwhelms the cell’s defenses. On the contrary, at a second region going much above 0.01 µ/mL, we observed a full reversion of effect. And in that region, a different mechanism from the first part takes place. Now, increasing the light dose decreases the antibiotic effect, indicated by the inversion of the order of the curves. With a high concentration of antibiotic, a higher light dose makes the result go farther from the no-photodynamic-addition. That indicates a full anti-synergic effect under some conditions, such as very low doses of light (5 J/cm^2^). PDT can induce sublethal stress, which paradoxically can trigger defense mechanisms in the bacteria, including the expression of efflux pumps that remove both ROS and antibiotics from the cell. This can reduce the efficacy of CIP, resulting in an antagonistic effect [19]. The presence of oxidative damage caused by PDT can trigger cellular responses that prioritize membrane and protein repair over DNA repair. If the bacterial cell preferentially allocates resources to repair damage caused by PDT rather than ciprofloxacin, a partial protective effect against the antibiotic may occur, attenuating the overall impact of the combination. The region between low dose and high dose of antibiotic indicates, however, a transition of behavior that we did not explore enough in this work but shall be explored in future studies. This inverse relationship in Figure 5 suggests that higher antibiotic concentrations reduce the need for energy exposure (such as UV light) to achieve antimicrobial effects. The data series demonstrates that the curves shift to the right with decreasing cell density, indicating that lower cell concentrations require lower antibiotic concentrations to achieve the same level of inactivation. This is consistent with the expectation that fewer cells require less energy or antibiotics to be inactivated. The series represented by 1 × 10^7^ CFU/mL (blue curve) requires the highest fluence to achieve equivalent levels of inactivation, while the series of 1 × 10^6^ CFU/mL (red curve) requires the lowest fluence. This suggests that the effectiveness of treatment with antibiotics or PDT significantly depends on the initial cell density, with denser populations being more challenging to inactivate.

Therefore, the combined efficacy of antibiotics with PDT strongly depends on the type of antibiotic and the PDT doses applied when all the other parameters are fixed. While gentamicin shows significant synergy with PDT, ceftriaxone does not benefit as much from this combination at the doses tested. On the other hand, CIP (ciprofloxacin) shows a complete inversion of behavior as the concentration of antibiotic increases. Previous studies have already demonstrated that exposure of *P. aeruginosa* to sublethal stress, such as low doses of PDT, can induce the activation of efflux pumps, such as the Resistance-Nodulation-Division family pumps, particularly MexAB-OprM, which are widely expressed in *P. aeruginosa* and play a key role in resistance to antibiotics, including fluoroquinolones such as ciprofloxacin. By generating ROS, PDT can cause an increase in oxidative stress, which acts as a signal for the activation of these pumps. This adaptive response allows the bacteria to expel both the antibiotic and the ROS generated by the treatment, reducing the effectiveness of both agents [20]. In addition to efflux pumps, the oxidative stress response generated by PDT can prioritize membrane and protein repair mechanisms, diverting cellular resources that would be used to repair DNA damage caused by ciprofloxacin. Ciprofloxacin acts by inhibiting the enzymes DNA gyrase and topoisomerase IV, which are essential for DNA replication and repair. However, suppose the stress caused by PDT is severe enough to cause oxidation of membrane components and important proteins. In that case, the bacterial cell can prioritize the repair of these structures over DNA, resulting in an attenuation of the antibiotic’s action [21]. These mechanisms may explain the antagonistic effect observed in high concentrations of ciprofloxacin combined with high doses of PDT. When the PDT dose is moderate or low, the oxidative stress generated may not be sufficient to trigger these adaptive responses, allowing greater cellular permeability and facilitating the action of ciprofloxacin. However, at higher doses, ROS production may reach critical levels that trigger robust cellular defenses, including the activation of efflux pumps and membrane repair systems, which antagonize the effect of the antibiotic. This phenomenon of antagonism between PDT and antibiotics has already been suggested by other studies, which indicate that the PDT dose should be carefully balanced to avoid the induction of undesirable adaptive responses [22].

This suggests that the choice of antibiotic and the optimization of the light dose are crucial to maximize the combined effect against *P. aeruginosa*. More than that, time may play an important role in the whole behavior. While the photodynamic dose has immediate action when applied, the antibiotic is time-dependent to start acting and continue to act. Therefore, we expect that the 6 h practiced in this experiment must be explored in shorter times and longer times. A very short waiting time after PDT may be dominated by photodynamic action alone due to the lack of time for antibiotic action. On the other hand, a much longer time may be fully dominated by antibiotic action, and the photodynamic action will be additive to the result. Their interference must occur at a medium time compatible with the time scale action of the antibiotic. Certainly, the universe of parameters to be explored is quite large. Optimal doses and exploring other combinations that can overcome the natural defenses of the bacteria and provide more effective treatments is a topic that requires much study since each microorganism and each antibiotic action has its specificity. In that sense, this study opened a new door of opportunities to tailor antibiotic effects with light. Our recent proposal to break the resistance of bacteria to the antibiotic with the application of PDT to let the antibiotic recover its bactericide power has been validated [Give ref to our PNAS papers] and needs further exploration in terms of its applicability to a wide range of antibiotics and treatment protocols.

The observation of synergistic and antagonistic effects between antibiotics and PDT was performed with a single bacterial strain, which is essential for standardizing the method. However, different bacterial species and strains may present varied responses due to genetic/phenotypic differences to antibiotic–PDT treatment. Performing standardization with other bacterial species may increase the application of this varied therapy, depending on the technology to be treated. Gentamicin disrupts bacterial protein synthesis by binding to ribosomal RNA, leading to protein misfolding. The generation of ROS by PDT may have enhanced this antimicrobial effect by increasing bacterial membrane permeability. Ceftriaxone, on the other hand, inhibits cell wall formation. With oxidative damage from ROS, the bacterial cell wall is weakened and may become more susceptible to ceftriaxone. Ciprofloxacin interferes with bacterial DNA replication, and ROS can generate DNA damage and aggravate this effect on DNA replication and repair. The results obtained by applying the Bliss independence model reinforce the qualitative observations presented throughout this study: (EB = EA + EP – EA × EP), where EB is the expected combined effect; EA is the isolated effect of the antibiotic; EP is the isolated effect of PDT; and EA × EP is the interaction between the effects of the antibiotic and PDT (both expressed as fractional inhibition, ranging from 0 to 1). E_obs_ is the actual experimental result, showing the combined effect provided directly in the experiment when applying both treatments. The synergy observed in the combination of gentamicin with PDT was confirmed quantitatively, with E_obs_ values consistently higher than the expected EB for all gentamicin concentrations and light doses. This suggests a strong synergistic interaction, possibly mediated by the increase in cell permeability induced by PDT, facilitating the action of the antibiotic. The results with ciprofloxacin revealed antagonism at higher concentrations, as qualitatively predicted. The Bliss model showed that E_obs_ was consistently lower than EB, especially at moderate doses of PDT. This behavior reinforces the hypothesis that bacterial defense mechanisms, such as the activation of efflux pumps, are contributing to the removal of ciprofloxacin and ROS generated by PDT, compromising the efficacy of the treatment. The precise quantification of the degree of antagonism provides a new dimension to understanding the limitations of the combination of ciprofloxacin and PDT and reinforces the need to optimize doses to avoid undesirable adaptive responses. The application of the Bliss model also suggests that the combination with ceftriaxone resulted in predominantly additive effects, with E_obs_ close to EB. Although PDT showed limited potential to increase the efficacy of ceftriaxone, the data suggest that adjustments in ceftriaxone concentration or light dose could lead to better therapeutic results. These observations highlight the importance of studying antibiotic and PDT combinations to find optimized protocols that maximize synergy and minimize antagonism.

## 3. Materials and Methods

### 3.1. Bacterial Cultures and Preparation

*P. aeruginosa* was purchased from the American Type Culture Collection (Manassas, VA, USA) (ATCC27853). A pre-inoculum solution was prepared according to a proportion of 1:9 of a cryotube bacteria sample (1 mL) and Brain Heart Infusion media culture (BHI) (9 mL) at pH 7.4 and incubated for 16 h and rotated at 150 rpm at 37 °C.

### 3.2. Antibiotic Preparation

We have used three antibiotics from different classes. Antibiotic powders were stored at 4 °C. Initially, a stock solution of 0.1 mg/mL was prepared in a sterile phosphate-buffered solution (PBS) (pH 7.4). According to the stock, the work solutions were diluted (Table 2) and stored in the dark at room temperature for further tests.

### 3.3. Antibiotic Incubation Protocol

The same protocol was followed for all antibiotic classes. The bacteria cells were washed twice with PBS, and the inoculum was standardized to an initial concentration of 1 × 10^7^ CFU/mL in a 10 mL solution. In a 24-well plate, 200 µL of the standardized inoculum was added to each well with 200 µL of each antibiotic solution diluted in different concentrations. Then, the cells were incubated with the antibiotics for 100 min in the dark at 37 °C. The control group was incubated with 200 µL of PBS. The groups were performed in triplicate.

### 3.4. PDT Protocol

#### 3.4.1. Photosensitizer

**Figure 6:**
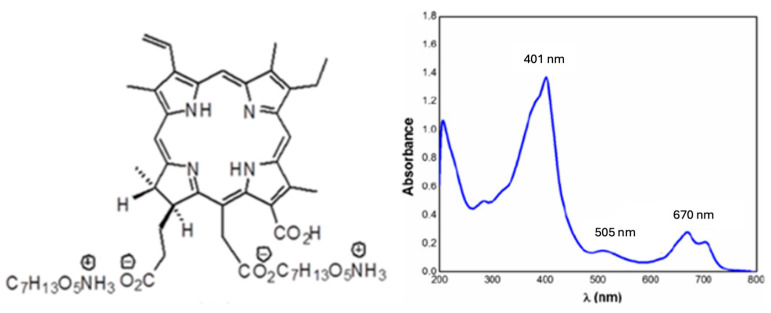
Molecular structure and absorption spectrum for the photosensitizer PDZ used in this study.

Photodithazine (PDZ) is a chlorine e-6 provided for us by the collaborative efforts of the University of Sao Paulo—Texas A&M University. A stock solution was prepared at 0.5 mg/mL, and from this, the work solutions were prepared using PBS. All the solutions were maintained in the dark at room temperature. The structure and optical absorption of the PDZ used are shown in Figure 6. The photosensitizer was chosen based on the experience of the group [23] and its efficacy in the treatment of Gram-positive and Gram-negative bacteria.

#### 3.4.2. Irradiation Device

The irradiation device was built by the Technical Support laboratory from the Physics Institute of São Carlos (USP/SP/Brazil). The equipment is composed of a plate holder composed of 24 LEDs with an irradiance of 50 mW/cm^2^, emitting homogeneously on the surface of the plates and operating at 660 nm. Reference to the device can be found in [24].

#### 3.4.3. PDT Application

**Figure 7:**
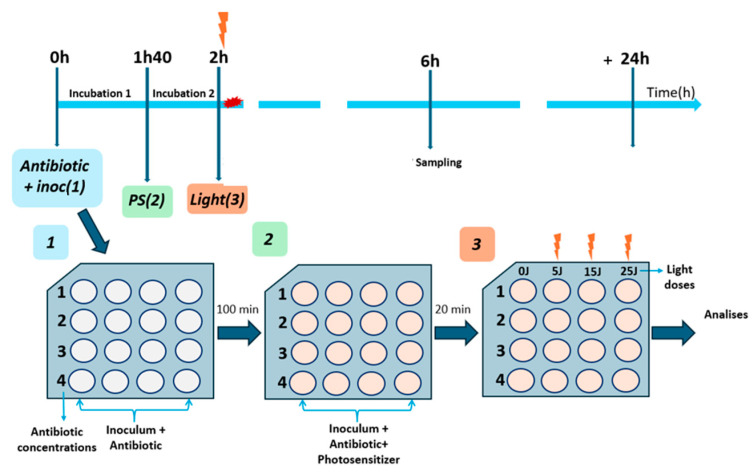
The methodology applied to first combine antibiotics with inoculum (1), then add PDZ solution at 0.01 mg/mL and incubate for 20 min (2), and then irradiate for a different time for different light doses (0, 5, 15, and 25 J/cm^2^) (3). The samples were performed in triplicate and analyzed at 6 h of the experiment.

After 100 min of the inoculum solution being incubated in a different concentration of antibiotic, 200 µL of PDZ solution with a concentration of 0.01 mg/mL (kept constant for all the experiments) was added to each well, and the plates were incubated for a further 20 min at 37 °C in the dark. The plate was irradiated by the irradiation device at different times, depending on the exposure of the system to different light doses. The light doses chosen were 5 (2 min), 15 (6 min), and 25 (10 min) J/cm^2^. Consecutively, each well was diluted by a serial dilution, plated in BHI agar, and incubated for 24 h at 37 °C for the colony-forming units (CFU/mL). In Figure 7, the overall time sequence of the methodology applied for the experiment is illustrated. In that sequence, the antibiotic acts for 6 h, and during that time, 2 h after the antibiotic, the photodynamic therapy was completed.

### 3.5. Statistical Analysis

The data were presented as means and SD (standard deviation). Two-group comparisons were performed by student’s *t*-test and using one-way ANOVA followed by Tukey’s test, considering two-tailed *p* values < 0.05 statistically significant. The analyses were performed using the software Origin^®^ version 10.5.119.52486 academic, with license granted by the University of Sao Paulo (USP).

## 4. Conclusions

In conclusion, this study highlights the importance of investigating in more detail the effects that originated during the combination of photodynamic therapy with antibiotics. Time sequence can be crucial to reach the final desired effect against *Pseudomonas aeruginosa*, one of the targets of our program of pneumonia treatment. As much as we believe that this may be a general phenomenon, there is a great degree of dependence related to microorganisms, antibiotics, and the concentration of photosensitizer used. In that sense, the results obtained may not be unique due to the large variety of possible conditions. Nevertheless, the interest in such a combination is large enough that even partial results like this have great receptivity in the literature.

## Figures and Tables

**Table 1:** Characteristics of the antibiotics ciprofloxacin (CIP), ceftriaxone (CEF), and gentamicin (GEN).

Ciprofloxacin (CIP)	Ceftriaxone (CEF)	Gentamicin (GEN)
Antibiotic Class: Member of 2nd-generation fluoroquinolone	Antibiotic Class: Member of 3rd-generation β-lactam cephalosporin	Antibiotic Class: Member of aminoglycoside
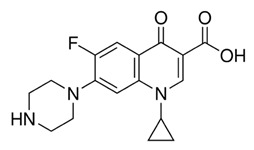	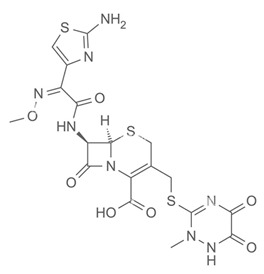	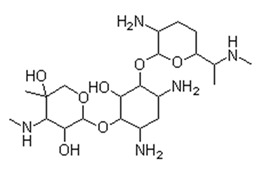
Properties: Melting point—255–257 °C; Water solubility—1.35 mg/mL.	Properties: Melting point—>155 °C; Water solubility—0.105 mg/mL.	Properties: Melting point—105 °C; Water solubility—12.6 mg/mL.
Brand Names: Cetraxal, Ciloxan, Cipro, Ciprodex, Ciprofloxacin, Otiprio, Otixal, Otovel, Proquin	Brand Names: Rocephin	Brand Names: Gentak, Pred-G, Valisone-G
Synonyms: Ciprofloxacin, Ciprofloxacine, Ciprofloxacino, and Ciprofloxacinum	Synonyms: Ceftriaxona, Ceftriaxone, Ceftriaxonum, and Rocephin	Synonyms: Gentamicin and Gentamicina
Mechanism of action: Inhibition of enzymes required for bacterial DNA replication, transcription, and repair.	Mechanism of action: Inhibition of mucopeptide (peptidoglycan) synthesis in the bacterial cell.	Mechanism of action: Bind to bacterial ribosomal RNA and inhibit protein synthesis.
Indication: Effective in patients with urinary tract infections, bone and joint infections, and lower respiratory tract infections.	Indication: Effective for the treatment of bacterial infections, pneumonia, ear infections, pelvic inflammatory disease, meningitis, skin infections, and gonorrhea.	Indication: Effective for the treatment of meningitis, infection of the blood, and serious urinary tract infections.
Affected bacteria: Gram-positive cocci, Gram-negative bacilli, MRSA, *Pseudomonas*, *Legionella*.	Affected bacteria: Gram-negative rods (except *Pseudomonas*). Gram-positive cocci (except methicillin-resistant and group D streptococci).	Affected bacteria: Aerobic Gram-negative bacteria—*Pseudomonas*, *Enterobacter*, *Proteus*, *Klebsiella*, etc. Gram-positive bacteria—*Staph*, *Strep*. No activity against anaerobes.
Metabolism: Primarily metabolized by CYP1A2	Metabolism: Negligible	Metabolism: Undergoes little to no metabolism

**Table 2:** Concentrations tested for each antibiotic.

Antibiotics	Concentration (µg/mL)
Ciprofloxacin	0.0001, 0.001, 0.01, 0.1
Ceftriaxone	0.001, 0.01, 0.1, 1, 1000
Gentamicin	1 × 10^−6^; 1 × 10^−5^, 1 × 10^−4^, 1 × 10^−3^, 1 × 10^−2^

## Data Availability

Data are contained within the article.
